# PBN (Phenyl-*N*-Tert-Butylnitrone)-Derivatives Are Effective in Slowing the Visual Cycle and Rhodopsin Regeneration and in Protecting the Retina from Light-Induced Damage

**DOI:** 10.1371/journal.pone.0145305

**Published:** 2015-12-22

**Authors:** Megan Stiles, Gennadiy P. Moiseyev, Madeline L. Budda, Annette Linens, Richard S. Brush, Hui Qi, Gary L. White, Roman F. Wolf, Jian-xing Ma, Robert Floyd, Robert E. Anderson, Nawajes A. Mandal

**Affiliations:** 1 Department of Ophthalmology, OUHSC, Oklahoma City, Oklahoma, United States of America; 2 Department of Physiology, OUHSC, Oklahoma City, Oklahoma, United States of America; 3 Department of Cell Biology, OUHSC, Oklahoma City, Oklahoma, United States of America; 4 Department of Pathology, OUHSC, Oklahoma City, Oklahoma, United States of America; 5 Department of Endocrinology and Diabetes, OUHSC, Oklahoma City, Oklahoma, United States of America; 6 Oklahoma Center for Neuroscience, OUHSC, Oklahoma City, Oklahoma, United States of America; 7 Dean McGee Eye Institute, Oklahoma City, Oklahoma, United States of America; 8 Experimental Therapeutics, Oklahoma Medical Research Foundation, Oklahoma City, Oklahoma, United States of America; Medical University of South Carolina, UNITED STATES

## Abstract

A2E and related toxic molecules are part of lipofuscin found in the retinal pigment epithelial (RPE) cells in eyes affected by Stargardt’s disease, age-related macular degeneration (AMD), and other retinal degenerations. A novel therapeutic approach for treating such degenerations involves slowing down the visual cycle, which could reduce the amount of A2E in the RPE. This can be accomplished by inhibiting RPE65, which produces 11-*cis*-retinol from all-*trans*-retinyl esters. We recently showed that phenyl-N-tert-butylnitrone (PBN) inhibits RPE65 enzyme activity in RPE cells. In this study we show that like PBN, certain PBN-derivatives (PBNDs) such as 4-F-PBN, 4-CF_3_-PBN, 3,4-di-F-PBN, and 4-CH_3_-PBN can inhibit RPE65 and synthesis of 11-*cis*-retinol in *in vitro* assays using bovine RPE microsomes. We further demonstrate that systemic (intraperitoneal, IP) administration of these PBNDs protect the rat retina from light damage. Electroretinography (ERG) and histological analysis showed that rats treated with PBNDs retained ~90% of their photoreceptor cells compared to a complete loss of function and 90% loss of photoreceptors in the central retina in rats treated with vehicle/control injections. Topically applied PBN and PBNDs also significantly slowed the rate of the visual cycle in mouse and baboon eyes. One hour dark adaptation resulted in 75–80% recovery of bleachable rhodopsin in control/vehicle treated mice. Eye drops of 5% 4-CH_3_-PBN were most effective, inhibiting the regeneration of bleachable rhodopsin significantly (60% compared to vehicle control). In addition, a 10% concentration of PBN and 5% concentration of 4-CH_3_-PBN in baboon eyes inhibited the visual cycle by 60% and by 30%, respectively. We have identified a group of PBN related nitrones that can reach the target tissue (RPE) by systemic and topical application and slow the rate of rhodopsin regeneration and therefore the visual cycle in mouse and baboon eyes. PBNDs can also protect the rat retina from light damage. There is potential in developing these compounds as preventative therapeutics for the treatment of human retinal degenerations in which the accumulation of lipofuscin may be pathogenic.

## Introduction

At present, approximately 1.75 million Americans have age-related macular degeneration (AMD) [1]. Diseases like AMD and Stargardt’s Disease (STGD), a juvenile form of macular degeneration, are particularly devastating because they destroy central vision and inhibit normal daily function. The atrophic, non-exudative, or drusenoid macular degeneration, collectively called 'dry AMD,' accounts for about 90% of all AMD cases. Dry AMD does not usually cause complete loss of vision, but significantly impairs central vision required for reading, driving, and other visually detailed tasks. A substantial proportion of advanced dry AMD transforms to 'wet' or neovascular AMD, which is vision threatening. Anti-angiogenic therapies have been developed for wet AMD, but there is no proven therapy for dry AMD [2].

One of the hallmarks of AMD and several other retinal degenerative diseases is the presence of lipofuscin in the retinal pigmented epithelium (RPE). One of the components of lipofuscin is A2E, a bisretinoid that is a product resulting from the condensation of all-*trans*-retinal with the membrane lipid phosphatidylethanolamine which has been studied in great detail for their role in RPE pathology [3–5]. All-*trans*-retinal is generated when the visual pigment rhodopsin absorbs a photon of light. All-*trans*-retinal is reduced by retinal dehydrogenases to all-trans-retinol and transported to the RPE where it is converted to all-*trans*-retinyl ester by lecithin retinol acyl transferase (LRAT) localized in the RPE cells. This is followed by conversion to 11-*cis*-retinol by RPE65, which is further oxidized to 11-cis retinal and then returned to the retina and incorporated into rhodopsin, thus completing the ‘visual cycle’. The excess of all-*trans*-retinal after rhodopsin bleaching can induce toxicity in photoreceptors at certain unfavorable conditions such as high intensity light or genetic mutations [6–8] in addition to the toxic effects of A2E and other bisretinoids.

With age, and in some inherited diseases, there is an accumulation of lipofuscin (and A2E) in the RPE. Previous studies demonstrated that A2E is toxic to RPE and other cells, and its accumulation in the RPE is thought to be one of the events that ultimately lead to the death of RPE cells [9–12].

One strategy currently being studied to reduce the accumulation of A2E in the RPE is to slow down the ‘visual cycle’, which will reduce the amount of 11-*cis*-retinal available for formation of rhodopsin, and thus reduce the amount of all-*trans*-retinal that is produced by light. The key step of the visual cycle is the cleavage-isomerization of all-*trans*-retinyl ester to 11-*cis*-retinol. Previous studies have established that the enzyme that catalyzes the isomerization step is RPE65 (retinal pigment epithelium-specific protein of 65 kDa) isomerase, which is expressed in the RPE [13–15]. Intact RPE65 function is essential for vision, as mutations in the RPE65 gene cause several forms of inherited retinal dystrophies [16–20].

Over 10 years ago, we discovered that phenyl-N-*tert*-butylnitrone (PBN), a spin-trap agent used for measuring the production of oxygen free radicals, protected the retina from light-induced retinal degeneration (LIRD) [21]. In *in vitro* enzyme assay, Poliakov et al., 2011, showed inhibition of RPE65 by PBN [22]. We performed a series of *in vivo* studies and witnessed PBN inhibition of RPE65 in rats [23]. We also demonstrated that PBN does not affect or inhibit the function of retinal dehydrogenases (RDHs) present in the photoreceptor outer segments and LRAT localized in the RPE cells [23]. PBN injected intraperitoneally in rats significantly affected the rate of regeneration of rhodopsin and recovery of the maximal a-wave response of the electroretinogram, consistent with a slowing of the visual cycle [23]. Under these conditions, there was no effect on the photoresponse of cones, indicating that the slowing down of the rod visual cycle in these animals did not affect the ability of their cones to respond to light.

Here we report the development and testing of certain PBN-derivatives (PBNDs) for their effect on the visual cycle and light-induced retinal degeneration. We also report the efficacy of PBN and PBNDs in slowing down the rate of rhodopsin regeneration and the visual cycle when applied topically to mouse and baboon eyes.

## Materials and Methods

### Animal care

All procedures were performed according to the Association for Research in Vision and Ophthalmology Statement for the Use of Animals in Ophthalmic and Vision Research. Albino Sprague-Dawley rats and BALB/c mice were born and raised in the Dean A. McGee Eye Institute vivarium and maintained from birth under dim cyclic light (5 lux, 12 h on/off, 7 a.m. to 7 p.m. CST). All the procedures, tissue harvest and the methods of euthanasia for mice and rats were reviewed and approved by the OUHSC Institutional Animal Care and Use Committee (OUHSC IACUC #12–063). Rats and mice were euthanized by carbon dioxide asphyxiation before harvesting the eye or retinal tissues.

Adult male and female baboons (*Papio anubis*) were obtained from the OUHSC Baboon Research Resource breeding colony and housed in an AAALAC-accredited vivarium at OUHSC. During the study, baboons were housed either individually or in pairs under regular indoor cyclic light (approximately 325 lux, 12 h on/off, 7 a.m. to 7 p.m. CST). They were fed a commercial diet of monkey chow along with fresh fruit and vegetables. Baboons were housed individually in Group 6 enclosures for nonhuman primates (84 inches in height, with 25.1 square-feet of floor space), or they were housed in pairs in two joined Group 6 enclosures (84 inches in height, with 50.2 square-feet of floor space) [cit: Code of Federal Regulations 2013 9 CFR §3.80]. Each baboon received social contact and structural, manipulable, and food enrichment in accordance with the OUHSC Baboon Research Resource Environmental Enrichment program. All animal procedures, including experiments and interventions, as well as euthanasia, were reviewed and approved by the OUHSC Institutional Animal Care and Use Committee (OUHSC IACUC #12–063).

### Materials

PBN was purchased from Sigma-Aldrich (St. Louis, MO) and the other derivatives were custom synthesized. Briefly, to synthesize 4-Fluorophenyl t-butyl nitrone (4-F-PBN), 4-fluorobenzaldehyde (8 mmol) and N-tertbutylhydroxylamine (120 mmol) were mixed in chloroform with molecular sieves (50 g, 4 A) and silica gel (10 g). The mixture was sealed under argon gas and stirred for 70 h at room temperature. The mixture was then filtered and the solid washed with ethyl acetate and the combined solution was rotary evaporated to give the crude product. The compound 4-trifluoromethylphenyl t-butylnitrone (4-CF_3_-PBN) was synthesized in a similar process of 4-F-PBN from 4-trifluoromethylbenzaldehyde and t-butylhydroxylamine. Similarly, 4-Methylphenyl t-butyl nitrone (4-CH_3_-PBN) was prepared by the reaction of 4-methylbenzaldehyde with t-butylhydroxylamine; 4-methoxyphenyl t-butylnitrone (4-CH_3_O-PBN was synthesized from 4-methoxybenzaldehyde and t-butylhydroxylamine; 4-Ethoxyphenylethoxyphenyl t-butylnitrone (4-EtO-PBN) from 4-ethoxybenzaldehyde and t-butylhydroxylamine; 2-fluorophenyl t-butylnitrone (2-F-PBN) from 2-fluorobenzaldehyde and t-butylhydroxylamine; 3-fluorophenyl t-butylnitrone (3-F-PBN) from 3-fluorobenzaldehyde with t-butylhydroxylamine; 3,5-difluorophenyl t-butylnitrone (3,5-diF-PBN) from 3,5-difluorobenzaldehyde and t-butylhydroxylamine; and 3,4-difluorophenyl t-butylnitrone (3,4-diF-PBN) from a reaction of 3,4-difluorobenzaldehyde with t-butylhydroxylamine. All of the compounds were purified by HPLC to >98% purity.

### *In vitro* assay for RPE65

The effect of several PBN analogs on RPE65 retinoid isomerase activity was investigated in an *in vitro* assay detailed in Mandal et al., 2011 [23]. Bovine RPE microsomes were used as a source of the RPE65 protein, and all*-trans*-[^3^H] retinol was a substrate for the retinoid isomerase assay. Although all-*trans*-retinyl ester was shown to be a direct substrate of the retinoid isomerase [24], very poor water solubility of retinyl ester limits its use in the *in vitro* retinoid isomerase assay. Therefore, all-*trans*-[^3^H]-retinol was used to generate retinyl esters in the microsomes of the bovine RPE, which is catalyzed by LRAT localized in RPE microsomes. Therefore, our assay tested activities of both LRAT and RPE65. The retinyl esters in RPE microsomes were then isomerized/hydrolyzed to 11-*cis*-retinol by the bovine RPE65. We previously showed that PBN specifically inhibits RPE65, not the RDHs and LRAT [23]. The produced 11-*cis*-[^3^H]-retinol was quantified by normal phase HPLC with flow scintillation analyzer.

### Intraperitoneal dosing of PBN derivatives in Sprague Dawley rats for rhodopsin measurement

PBNDs 4-F-PBN, 4-CF_3_-PBN and 4-CH_3_-PBN were dissolved in saline with saline alone used as vehicle. PBNDs and vehicle were injected intraperitoneally into light-adapted (200–400 lux room light) rats. The light source for this bench top light adaptation was cool white light bulbs (13w, F13T5/CW from Sylvania s088). The rats were kept in ambient light for 2h and then moved into total darkness for 2.5h, after which these dark-adapted rats were euthanized and retinas were immediately harvested under dim red light. Dark adapted-controls were rats that experienced dark adaptation for 12h and received no treatment and no light exposure before euthanasia, as described above, followed by retinal harvest. Light adapted-controls were rats that experienced ambient light (200–400 lux) for 2h and received no treatment before euthanasia followed by retinal harvest in light. Rhodopsin measurement was conducted following the classical method of David Papermaster [25] with slight modification that we published earlier [21,23,26,27]. Briefly, under red light, each retina was homogenized in 450 μl of buffer containing 10 mM Tris-HCl (pH 7.4), 150 mM NaCl, 1 mM EDTA, 2% (w/v) octyl glucoside, and 50 mM hydroxylamine for 1 minute. Hydroxlyamine is used to rapidly form a stable oxime with all-*trans*-retinal that is produced by the bleaching of rhodopsin. This complex absorbs around 360 nm, which is further into the UV spectrum than the bleaching intermediates of rhodopsin and its more stable product meta-II (380 nm). This oxime guarantees that the difference spectrum (native vs. bleached rhodopsin) at 500 nm will not be contaminated with the bleaching intermediates. Homogenates were centrifuged at 16,000 × g, and soluble lysates were scanned from 270 to 800 nm in a spectrophotometer (Ultrospec 3000 UV-visible spectrophotometer, GE Healthcare). Samples were then bleached under room light (200–400 lux) for 10–20 min and scanned again. The difference in spectra at 500 nm between pre- and post-bleached samples was used to determine rhodopsin content using a molar extinction coefficient of 42,000 M^−1^ [28]. The rhodopsin content was calculated as picomoles/retina.

### Light-damage of the retina following dosing of PBNDs

PBNDs were dosed following the same dosing scheme as rhodopsin measurement. After dosing, SD rats were placed in dim-light conditions for 30 min before exposure to 2,700 lux white reflected light from white electric bulb (32w, F32T8/TL84, Philips Long Life Plus) fitted on the top of a specially fabricated light box. All the sides and the bottom of the box are painted white. Rats are housed single in wire-top transparent plastic cages with the water bottle from the side not to block light for 6h of exposure. There is approximately 3 feet distance between the light source and the rats and there is ample air circulation in the box to prevent heating up. The lux level is measured inside the cage at the level of the rats’ eyes. After exposure, the rats were returned to a dim-light cyclic room (5–10 lux) to recover for 7 days. Retinal damage/protection was assessed by functional analysis using ERG and structural analysis of histological sections following the procedure published previously [23,26,29,30].

### Topical administration of PBNDs in BalbC mice for rhodopsin measurement

For topical administration of PBN, a 10% concentration was prepared in 1x phosphate-buffered saline, (pH 7.4, PBS) with 30% 1-Methyl-2-pyrrolidinone (MPA) used as a solubilizer. PBNDs were made to a 5% concentration in PBS using 15% MPA. BalbC mice raised in dim light conditions (5–10 lux) were dark-adapted overnight. The following morning the mice were moved to ambient room light (200–400 lux) and given 1 drop of the PBN solutions in each eye once every 3 hours over a 6 hour period resulting in 3 total doses before returning to the dark for 1 hour. The mice were euthanized by CO_2_ asphyxiation in the dark followed by whole eye harvest for rhodopsin measurement. 30% MPA in PBS was used as PBN vehicle control. For the PBNDs 15% MPA in PBS was used as vehicle control. Dark adapted no treatment controls were used as a baseline for 100% rhodopsin. Light adapted no treatment controls were used to verify 100% rhodopsin photo bleaching. Rhodopsin was measured as described above and the rhodopsin content calculated as picomoles/eye.

### Topical administration of PBN and 4-CH_3_-PBN in baboons to measure rhodopsin regeneration

A total of 12 baboons used for this study (4 for PBN and 8 for 4-CH3-PBN). All baboons used for this study were scheduled for euthanasia as determined by the OUHSC Attending Veterinarian for reasons unrelated to this study. No baboon died without euthanasia. The baboons were evaluated at least twice daily by clinical veterinarians and a staff environmental enrichment coordinator to ensure that humane endpoints were observed and animals did not experience pain or distress. Once the decision was made to euthanize an animal, the veterinary staff promptly notified the investigator and the acute procedure was performed under the approval of the Attending Veterinarian. Reasons for scheduled euthanasia included chronic illness that was nonresponsive to therapy (e.g., idiopathic epilepsy, irritable bowel syndrome, septic shock), or social incompatibility with demonstrated aggression despite multiple efforts to habilitate animals to a colony environment. Other factors including participation in previous experimental procedures were also taken into account for euthanasia criteria. The baboons were evaluated at least twice daily by clinical veterinarians and a staff environmental enrichment coordinator to ensure that humane endpoints were observed and animals did not experience pain or distress until euthanasia occurred. Baboons were fasted 12 hours prior to the procedure. On the day of the procedure, each animal was chemically restrained using Ketamine (10mg/kg IM). Once immobile, Propofol (4mg/kg IV) was administered to induce anesthesia. Anesthesia was maintained using isoflurane (1–2%). Blood pressure, pulse oximetry, heart rate, respiratory rate, body temperature, and anesthetic depth were monitored throughout the duration of the procedure. Baboons were dosed in the left eye with 1 drop of either 10% PBN or 5% 4-CH_3_-PBN every 15 minutes over a 2h period (total of 8 doses). Vehicle was applied to the right eyes using the same schedule. Two hours after the first drops, Euthasol (1mg/5kg IV) was administered for euthanasia. The eyes were thoroughly flushed with vehicle, enucleated, and rinsed again with vehicle. Eyes were wrapped in aluminum foil and placed on ice for 2 hours, after which each eye was hemisected under dim red light and the anterior segment discarded. The retina was dissected from the retinal pigment epithelium and suspended in a petri dish containing PBS. Four pieces of tissue from each retina were removed (still under dim red light) and placed into individual plastic tubes for rhodopsin measurement as described above. Protein values were determined by absorbance at 280 nm (for 1 cm path length cuvette, we considered 1 absorbance = 1 mg/ml protein) and the photobleachable rhodopsin content calculated as picomoles/mg protein. The percent regeneration in PBN-treated eyes was determined relative to values for the vehicle treated eyes, which were set at 100%.

### Statistical analyses

Statistical analyses were performed by using GraphPad Prism 5.0 software (GraphPad Software Inc., La Jolla, CA). The quantitative data are expressed as mean ± standard error of the mean (SEM) for each group. An unpaired Student t test was performed for the means of two unmatched groups; for three or more groups, one-way ANOVA was used to compare each pair of the test groups.

## Results

### Synthesis of new PBN Derivatives and their inhibition of RPE 65

**Fig 1:**
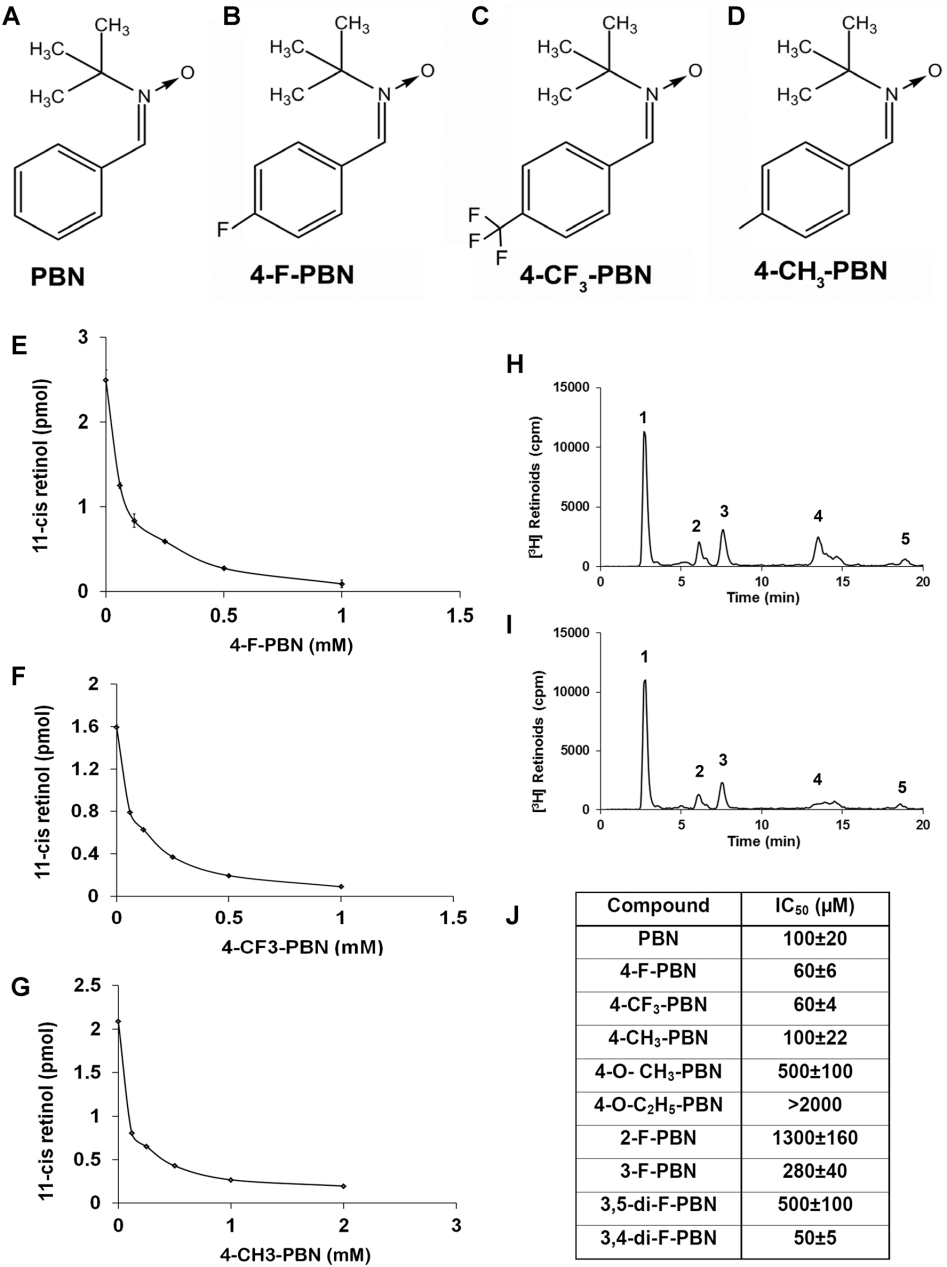
PBND tested for RPE-65 enzyme inhibition efficiency. The structure of PBN and the derivatives of PBN that are used in various *in vitro* and *in vivo* assays are shown in Fig 1A–1D. Concentration-dependent inhibition of RPE65 and generation of 11-*cis*-retinol in *in vitro* microsome assays were performed with representative images shown in Fig 1E, 4-F-PBN; Fig 1F, 4-CF_3_-PBN; and Fig 1G, 4-CH_3_-PBN. A table of the IC_50_ values of the compounds tested for their inhibition efficiency for RPE 65, (Fig 1J), show various inhibition rates for which we determined which PBN-derivatives looked the most promising to continue our analysis.

Working with a custom synthesis company, we synthesized nine derivatives of PBN that includes various fluorine-derivatives (4-F-PBN, 4-CF_3_-PBN, 2-F-PBN, 3-F-PBN, 3,4-di-F-PBN, and 3,5-di-F-PBN), methyl derivatives (4-CH_3_-PBN, 4-O-CH_3_-PBN) and an ethyl derivative (4-O-C_2_H_5_-PBN). We have tested three of these derivatives (4-F-PBN, 4-CF_3_-PBN, and 4-CH_3_-PBN) along with PBN in various *in vivo* assays to determine their efficacy in rhodopsin regeneration and protection of retina from light-induced damage (Fig 1A–1D). This selection was based on their efficacy in inhibiting RPE65 activity *in vitro* in a bovine microsome assay as described in the Materials and Methods section. We calculated IC_50_ values for all the derivatives that inhibit 50% of the enzymatic activity. The inhibition of retinoid isomerase activity was dependent on the concentration of the PBNDs (Fig 1E–1G; the enzyme inhibition graph is not shown for the other derivatives). Although all-*trans*-retinyl ester is the endogenous substrate for the RPE65 retinoid isomerase, the insolubility of retinyl ester in water prevented its direct use in the reaction. Therefore, all-*trans*-[3H] retinol was used to produce retinyl esters by LRAT in bovine RPE microsomes; these esters were then converted directly to 11-cis-[3H] retinol by RPE65. Incubation of the bovine RPE microsomes with all-*trans*-[3H] retinol resulted in formation of all-*trans*-retinyl esters and significant amounts of 11-*cis*-[3H] retinol, as shown by the HPLC elution profile (Fig 1H, representative for 4-CF_3_-PBN). The addition of 250 μM of 4-CF_3_-PBN to the retinoid isomerase assay reaction resulted in a significant inhibition of 11-*cis*-retinol production (peak 4), as shown by HPLC (Fig 1I), whereas the generation of retinyl ester (peak 1) was not decreased, suggesting that LRAT was not inhibited by 4-CF_3_-PBN. Some amounts of all-*trans*-retinal (peak 3) and 13-*cis*-retinal (peak 2) were observed due to the oxidation and thermal isomerization of all-*trans*-retinol (Fig 1H and 1I). Fig 1J represents a table of all the derivatives we tested for RPE65 activity assay. The fluorinated analogs 4-F-PBN, 4-CF_3_-PBN, and 3,4-di-F-PBN were the most efficient inhibitors. 4-CH_3_-PBN proved as effective as PBN.

A representative HPLC tracing of inhibition of the retinoid isomerase activity by 4-CF_3_-PBN is shown in Fig 1H and 1I. Bovine RPE microsomes (20 μg) were incubated with 0.2 μM of all-*trans*-[^3^H] retinol in the presence or absence of 4-CF_3_-PBN for 2 h at 37°C. The retinoids generated were analyzed by HPLC and flow scintillation counter. Fig 1H, HPLC elution profile without inhibitor; and Fig 1I, with 250 μM of 4-CF_3_-PBN. Fig 1H and 1I: Peak 1, retinyl esters; 2, 13-*cis*-retinal; 3, all-*trans*-retinal; 4, 11-*cis*-retinol; 5, all-*trans*-retinol.

### Effect of systemic administration of PBNDs on rhodopsin regeneration in rats

**Fig 2:**
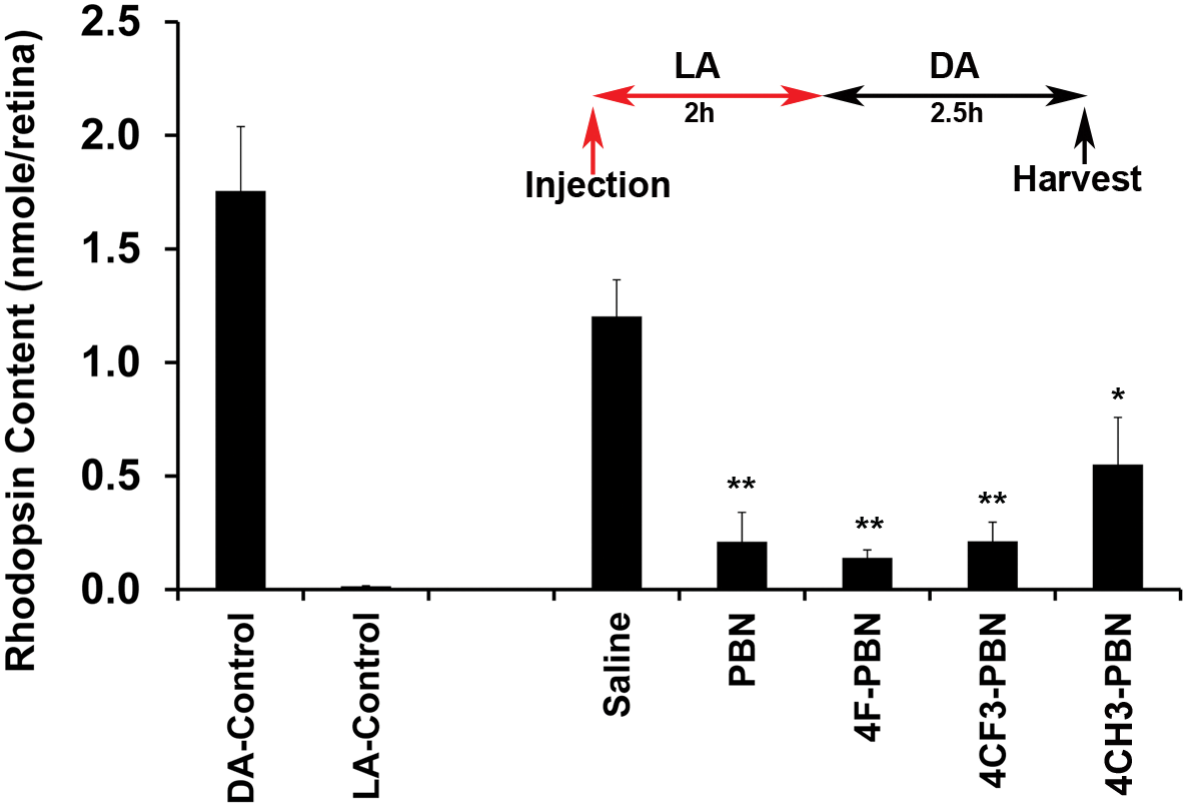
PBN and certain PBNDs inhibited rhodopsin regeneration. Rats were treated with either PBN or certain PBNDs before 2h of light adaptation followed by 2.5h of dark adaptation after which the animals were euthanized and retinas harvested for rhodopsin assay. Saline treated retina recovered ~ 75% rhodopsin in 2.5h dark. PBN and its derivatives, however, blocked rhodopsin regeneration significantly. LA, light adapted; DA, dark adapted. [* = *p*<0.01; ** = *p*<0.001; *n* = 4–6]

To determine if PBNDs delayed dark adaptation, we gave IP injections of our PBNDs (50 mg/kg) to light-adapted rats and kept them in the light for two additional hours, after which they were placed in total darkness for 2.5 h. Eyes were harvested under dim red light and rhodopsin content determined spectrophotometrically. As shown in Fig 2, rhodopsin regeneration was significantly reduced by administration of all PBNDs, compared to controls, which had 75% recovery of fully dark-adapted values. These results demonstrate profound inhibition of rhodopsin recovery by systemic administration of PBN and PBNDs.

### PBNDs protect retina from light stress

**Fig 3:**
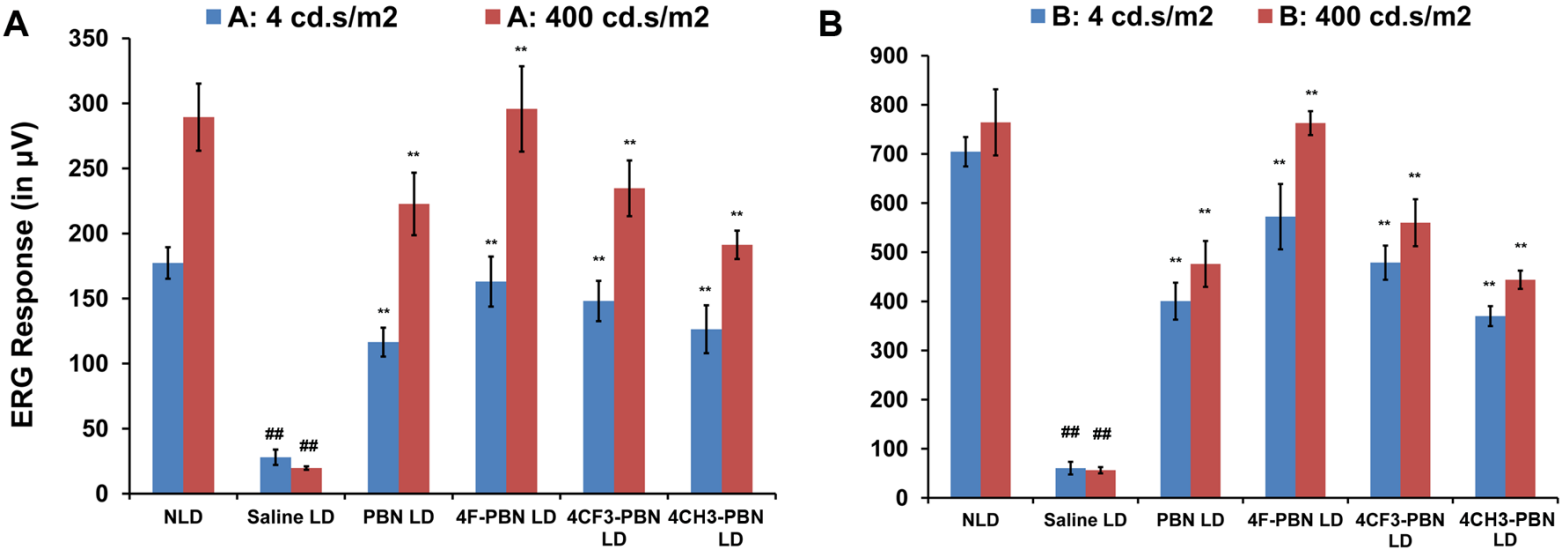
Systemically administered PBN and PBND protect rat retina from light damage. Electroretinographic (ERG) responses were recorded for a series of flash stimuli intensities at 0.04, 4, 200, 400, and 2000 cd.sec/m^2^. ERG responses presented here are from the intensities of 4 and 400 cd.sec/m^2^. The dim flash (4 cd.sec/m^2^) stimulates the rod photoreceptor cells and the bright flash (400 cd.sec/m^2^) stimulates both rod and cone photoreceptors. Therefore, the blue bars represent only rod responses and the red bars represent mixed response from both rod and cone photoreceptors. Fig 3A shows A-wave responses, and Fig 3B shows B-wave responses. NLD represents non-light-damaged group and LD represents light-damaged groups. [## = *p*<0.001: NLD vs. LD saline-treated; * = *p*<0.01 and ** = *p*<0.001: LD saline vs. PBNDs; *n* = 4–6]

In previous studies, we have shown that 50 mg/kg systemic PBN administered intraperitoneally 0.5 h before light stress (2,700 lux for 6 h) provided significant (80–90%) protection of retinal structure and function in rats [23]. Here, using similar parameters, we tested PBNDs in rats and compared their effects with PBN and saline (vehicle). ERGs were done 7 days after light exposure, after which eyes were harvested for histological analyses. ERG responses were significantly reduced in vehicle-treated rats after light stress (saline LD), whereas PBNDs significantly reduced the loss of ERG responses. All PBNDs were comparable to or better than PBN in preventing ERG losses (Fig 3A and 3B).

**Fig 4:**
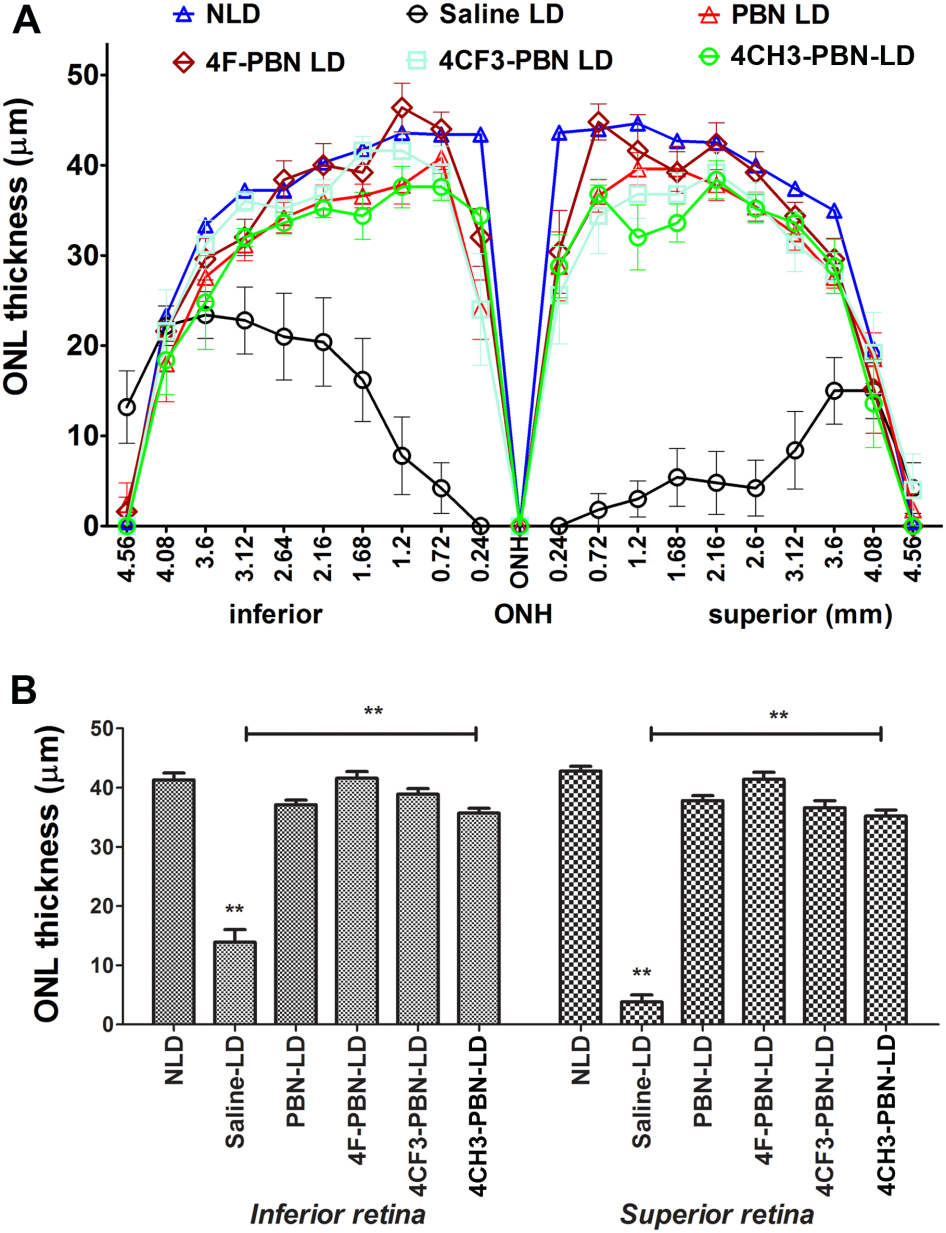
Histological analysis of rat retina confirms protection from light damage. Vehicle or Saline treated (Vehicle LD) rats lost most of their photoreceptors from the central retina. Effect is more pronounced in the superior retina. NLD is non-light-damage control. Fig 4A shows the ONL thickness across the retina in the vertical meridian. PBN treatment (PBN LD) retinas retained ~90% of photoreceptor cells. 4-CF_3_-PBN is comparable to PBN in protection, whereas 4-F-PBN appears better than PBN. 4-CH_3_-PBN also protects the retina significantly, although its effect is slightly less than PBN. Fig 4B shows central retinal outer retinal layer (ONL) thickness in both inferior and superior retina. [** = *p*<0.001; *n* = 8–10]

The findings obtained by functional ERG analysis were confirmed by quantitative morphometry of the thickness of the ONL of the retina. By 7 days, the dead photoreceptors were removed and the remaining nuclei in the ONL represent the viable photoreceptors. The spidergram in Fig 4A shows a thickness map of the retina in cross section through the vertical meridian. The light stress paradigm caused significant loss of photoreceptor cells, which was more pronounced in superior central retina. Pre-treatment with systemic PBN or PBNDs resulted in retention of most of the photoreceptor cells (Fig 4A). The average ONL thickness in the superior and inferior retina is shown in Fig 4B. PBN and all PBNDs significantly protected rod photoreceptors from light stress-induced degeneration. The protective effect of 4-F-PBN and 4-CF_3_-PBN were comparable to PBN, whereas the effect of 4-Me-PBN was slightly lower than that found for PBN (Fig 4A and 4B). Representative histological images of the retina are shown in (S1 Fig).

### Topical administration of PBNDs inhibits rhodopsin regeneration

**Fig 5:**
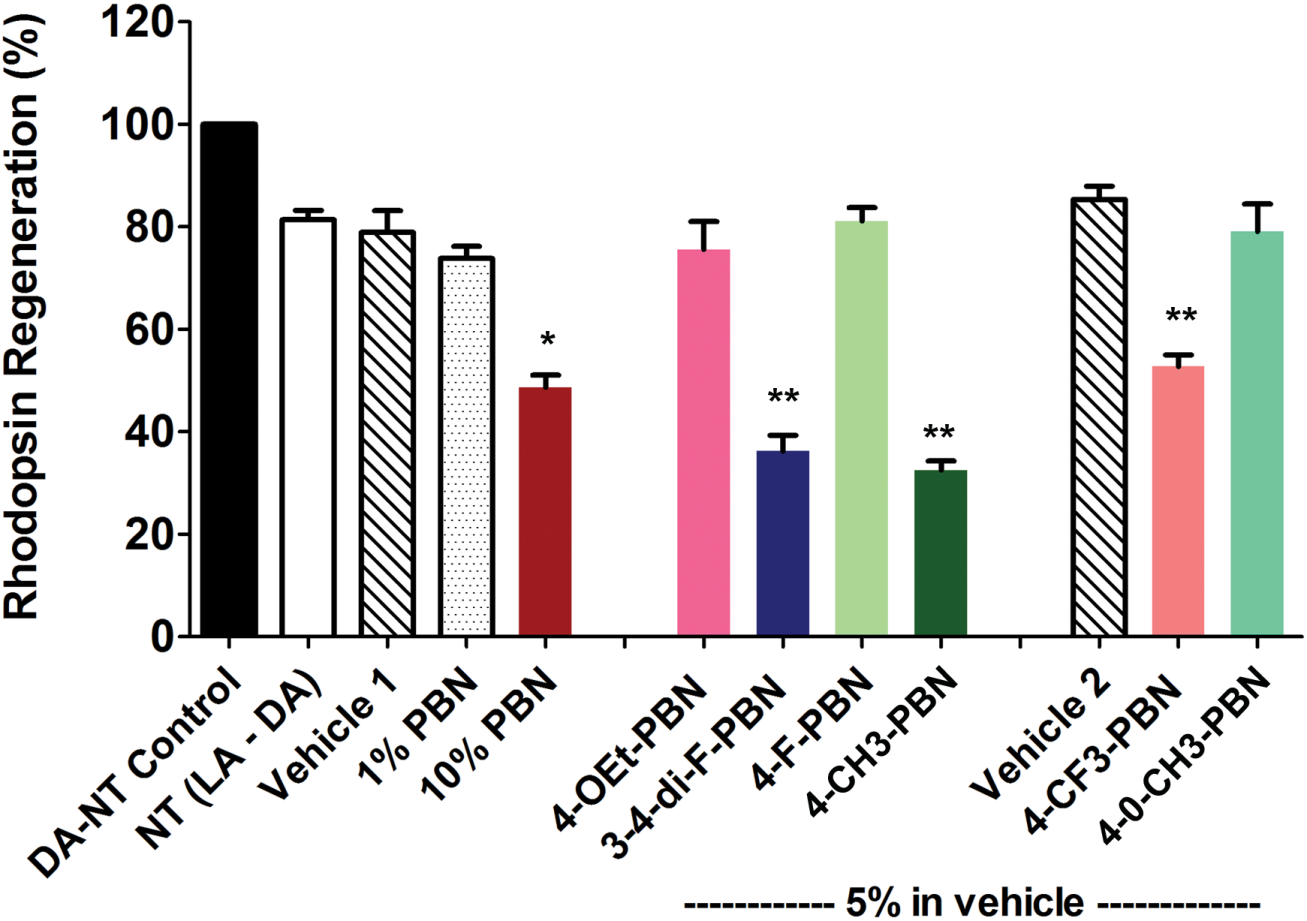
Mice treated topically with PBN or PBND’s showed reduction in rhodopsin regeneration. Dark adapted no treatment (DA-NT), no treatment light-adapted followed by dark adapted (NT LA-DA), and vehicle controls were used. Both 1% and 10% PBN were tested against 5% concentrations of PBNDs. Topical administration of assigned drug consisted of 1 drop in each eye once every 3 hours over a 6 hour period resulting in 3 total applications in light adapted mice eyes. Immediately following the 3^rd^ and final dose mice were placed in the dark for 1h before harvest. LA, light adapted; DA, dark adapted. [* = p<0.01; ** = p<0.001; n = 6]

To determine if any of the PBNDs can reach the retina following topical application (specifically to the RPE-choroid) and inhibit rhodopsin regeneration, we designed eye-drop formulations with six PBN derivatives as presented in Fig 1. Based on their IC50 values, we predicted that compounds with lower IC50 values would inhibit RPE65 enzyme activity to a greater extent, which would be evidenced by lower levels of rhodopsin regeneration after photobleaching. We applied 3 topical drops over a period of 6h in completely light-adapted mice with bleached rhodopsin (0% expected level). After the last eye-drop the mice were moved to dark for 1h to allow rhodopsin regeneration. One hour dark adaptation allowed 75–82% recovery of bleachable rhodopsin in untreated and vehicle-treated mice. A 10% PBN eye-drop inhibited rhodopsin regeneration significantly, whereas 1% PBN showed little inhibition (Fig 5). Presence of PBN in the RPE-choroid tissue was determined by mass spectrometry (S2 Fig). In addition, three other derivatives, 4-CF_3_-PBN, 3,4-di-F-PBN, and 4-CH_3_-PBN at 5% concentration also showed an inhibiting effect on rhodopsin regeneration with the greatest inhibition by 4-CH_3_-PBN (Fig 5).

### Topical administration of 10% PBN vs 5% 4-CH_3_-PBN in baboon eyes

**Fig 6:**
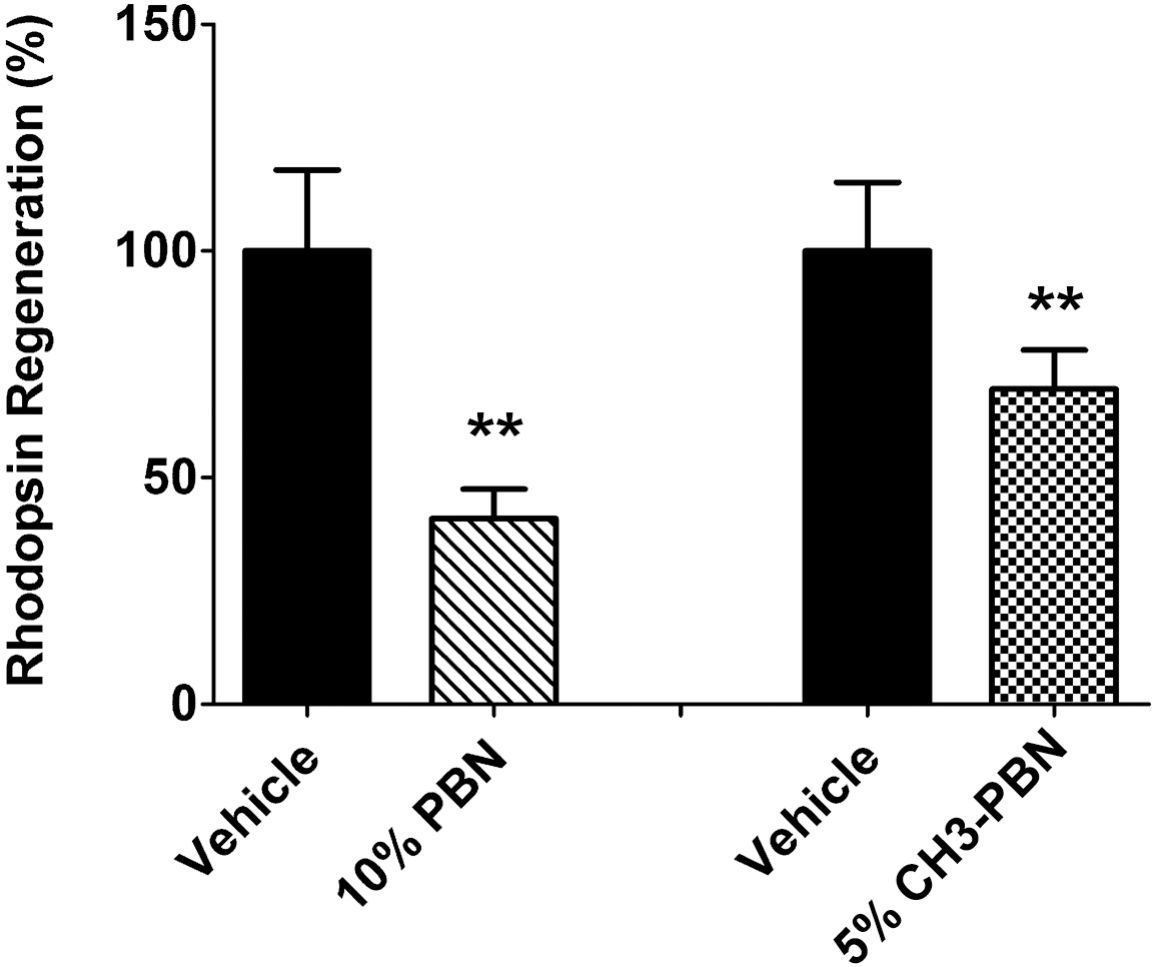
PBN and 4-CH_3_-PBN topically administered in baboon eyes slows rate of rhodopsin regeneration. Baboon eyes were treated topically with drug or vehicle every 15 minutes over a 2h period before eyes were harvested and dark adapted 2h before retinal harvest. 10% PBN showed a roughly 50% higher inhibition rate compared to 5% CH_3_-PBN suggesting both drugs to be effective. [** = *p*<0.001; *n* = 6–8 eyes]

With 4-CH_3_-PBN showing the greatest inhibition of rhodopsin in our mouse models, it was selected for study in baboon eyes alongside 10% PBN. Using the same formulation used in mice, 10% PBN and 5% 4-CH_3_-PBN were used for dosing anesthetized baboon eyes over a 2 hour period before harvest for rhodopsin measurement. PBN determinations in baboon eyes clearly show that the compound reached the RPE/choroid tissue (S1 Table). Rhodopsin regeneration was significantly lower in the eyes treated with 10% PBN compared to controls (Fig 6). These results support our finding in mouse eyes treated with PBN, namely that the drug reaches the RPE and inhibits the enzymatic activity of RPE65. 5% 4-CH_3_-PBN exhibited a similar effect to PBN in inhibiting rhodopsin regeneration when administered as an eye drop (Fig 6).

## Discussion

The goal for developing any kind of therapy for the treatment of macular degeneration should target the underlying cause of the disease and halt or slow the loss of vision. The accumulation of fluorescent materials such as lipofuscin in the RPE cells of the aging retina, which is most pronounced in the macula, induces RPE cell dysfunction and death in AMD and Stargardt’s patients. One strategy for protecting RPE cells is to develop drugs that slow down the normal visual cycle. The rationale for this approach is that the visual cycle produces all-*trans*-retinal in the photoreceptors, which serves as one of the precursors for the biosynthesis of A2E (a pyridinium bis-retinoid that is derived from two molecules of all-*trans*-retinal and one molecule of ethanolamine), a key component of lipofuscin [3–5]. These toxic retinoids can generate free radicals upon light exposure that can damage RPE cell membranes [10], inhibit RPE lysosomes (which leads to drusen formation) [9], and activate complement factors [12], a major genetic risk factor for AMD [31–34]. Therefore, inhibiting or slowing down the visual cycle should reduce the accumulation of A2E in the RPE cells.

We recently showed that PBN, when delivered systemically, can substantially slow down the visual cycle by inhibiting RPE65 [23]. In this study, we investigated the effect of several PBN analogs in both *in vitro* and *in vivo* assays to determine their effectiveness. Since PBN is a widely studied nitrone, we decided to explore the potential that modifications of the chemical structure may reveal increased understanding of its interaction with the active site and perhaps find a derivative that may have enhanced activity. Since specific PBN derivatives have been shown to have potent activities in other pre-clinical models [35], we first chose to test: A) the 4-hydroxyl-phenyl derivative of PBN (4-OH-PBN), which has been shown to be active in preventing noise-induced hearing loss [36], and B) the 2,4-disulfonyl-phenyl derivative of PBN (DS-PBN) which is seen to be active in pre-clinical models of acute ischemic stroke [35,37] and in models of glioma [38,39]. We found that 4-OH-PBN as well as DS-PBN had very little if any activity in slowing down the visual cycle in our previous studies [23]. Since the 4-hydroxyl group as well as the di-sulfonyl groups attached to the phenyl moiety of PBN represents major perturbations to the phenyl chemical structure and to its electronic nature, we rationalized that the intact phenyl group was very important and that other more minor chemical and electronic density modifications to its chemical structure may turn out to be informative. Working with a custom synthesis company we had them synthesize various fluorine-derivatives and methyl derivatives of the phenyl group of PBN and tested their activity in both *in vitro* and *in vivo* assays to determine their effectiveness in slowing the visual cycle by inhibiting RPE65 retinoid isomerase activity (Fig 1).

*In vitro* assays evaluated the inhibitory effect of PBN analogs on RPE65 retinoid isomerase in microsomal preparation of bovine RPE cells, whereas *in vivo* assays were carried out in rats, mice and baboons. The rat studies involved delivery of the compounds intraperitoneally, whereas the mouse and baboon *in vivo* studies were carried out by topical application of some selected compounds in the form of eye drops. In all *in vivo* studies, the activity of RPE65 was measured indirectly by rhodopsin regeneration. Inhibition of RPE65 will inhibit the formation of 11-*cis*-retinal in the RPE cells, which will lower its availability for regeneration of rhodopsin in rod photoreceptors. After treatment with PBN and PBNDs in the light (with fully bleached rhodopsin), the mice or rats were moved to the dark room for a specified time period to recover bleachable rhodopsin. Baboon eyes, on the other hand, were harvested in the light and wrapped in aluminum foil and stored at 4°C for 2 hours to allow recovery of rhodopsin, similar to what is done with porcine or bovine eyes obtained at an abattoir. In all animals tested in this study, PBN and PBNDs reduced the recovery of photobleachable rhodopsin when applied either by IP injection or topically.

The chemical structures of the PBN analogs that inhibited RPE65 activity similar to or more than PBN are shown in Fig 1. The fluorinated analogs 4-F-PBN, 4-CF_3_-PBN, and 3,4-di-F-PBN were the most efficient inhibitors. These three analogs were more effective at inhibiting RPE65 activity than the native PBN molecule. The IC_50_ values of 4-CH_3_-PBN were similar to PBN (Fig 1). Therefore, in search of more effective nitrones to inhibit RPE65, we found a few fluorinated derivatives that were superior to PBN. *In vivo* studies involving these derivatives, however, required tests concerning their delivery to determine bioavailability in the target tissue and inhibition of RPE65 in the eye.

In our previous studies, we tested PBN by systemic administration intraperitoneally [23]. Systemic PBN can inhibit rhodopsin regeneration and protect retina from light-induced damage. We therefore tested the new derivatives systemically for their effect on these parameters. Interestingly, the PBN analogs that were effective in inhibiting RPE65 *in vitro* were also effective *in vivo* (Figs 1, 2, 5, and 6). IP injections of these compounds with similar doses of PBN also prevented intense light-induced damage of the retina (Figs 3 and 4). We thus identified and verified three new compounds that can inhibit RPE65 *in vivo*, slow-down rhodopsin regeneration and, if administered prior to intense light exposure, can prevent retina from photo-damage. This could prove significant in terms of drug discovery; these compounds can be formulated for human use in cases where slowing rhodopsin regeneration would have therapeutic benefits.

Regeneration of rhodopsin during light exposure is essential for intense light-mediated damage of photoreceptor cells [40–42]. Regeneration of rhodopsin depends on the availability of 11-*cis*-retinal. Accordingly, vitamin A-deficient rats, rhodopsin knock-out (KO) mice that lack the opsin apoprotein, and RPE-65 KO mice that have opsin apoprotein but lack the ability to generate 11-*cis*-retinal are all protected against light damage [40,41,43–45]. RPE65 is one of the key enzymes of the visual cycle. It is the only retinoid isomerase in RPE cells and it catalyzes the rate-limiting step of the visual cycle converting all-*trans*-retinyl esters to 11-*cis*-retinol. Inhibition of RPE65 with a competitive inhibitor such as 13-*cis*-retinoic acid or retinylamine provides efficient protection of the retina from light induced damage [46–48]. RPE65 is therefore a potential target for slowing down the visual cycle. One of the strategies for treating blinding eye diseases that accumulate excessive undigested materials, including visual cycle byproducts in the RPE cells, is to slow the rod visual cycle, which is not required for vision in ambient light and can potentially reduce the accumulation of the byproducts such as the retinal fluorophore A2E, a component of lipofuscin [49]. We previously showed that the amount and dosing schedule of PBN we used in rats affected only rod function [23]. Our therapeutic goal is to slow the visual cycle during the daylight hours when rod vision is not needed. Because of the short half-life (~2 h) of PBN in the rat retina [50], we anticipate that dosing only during the day would allow sufficient time for PBN to be cleared before rod function would be needed, thus avoiding the possibility of chronic night blindness. Also, we do not need to inhibit ‘rod visual cycle’ completely; a dose of day time nitrones that inhibits 30–40% RPE65 could be proved effective in the long term in reducing accumulation of A2E. Therefore, the PBN derivatives we tested herein could potentially be developed as therapeutics for those retinal degenerations in which accumulation of A2E leads to pathogenesis.

Our ultimate goal is to develop a human drug for Stargardt’s Disease and dry-AMD therapies. The method of delivery is thus quite salient. A topical application will have significant advantages over systemic (oral) or intravitreal delivery due to a reduced systemic effect and/or ease of application for a prolonged period of time. We therefore tested topical application of the PBN derivatives in mice to determine their bioavailability and effect on RPE65, which was measured by rhodopsin regeneration. We found that 4-CH_3_-PBN was the most effective through topical application followed by 3,4-di-F-PBN. PBN and 4-CH_3_-PBN were further tested in larger mammal (baboon) eyes and were found to reach the RPE and effectively reduce rhodopsin regeneration.

In summary, we have identified novel nitrones that can inhibit RPE65 enzyme activity *in vitro* and *in vivo*. Some of these nitrones can be delivered topically in mice and baboons, which suggests a greater opportunity for developing therapeutic drugs for human retinal degenerations based on molecular nitrones.

## Supporting Information

S1 Fig Representative images of H&E sections of rat eyes treated with PBNDs and light damaged. After ERG recordings, eyes we enucleated for histology, marked for orientation, and fixed. Five-μm sections were cut along the vertical meridian through the optic nerve and stained with H&E. Representative sections from each treatment were imaged from the superior-central retina, which is specifically affected by exposure to damaging light. A, no light damage control (NLD); B, no treatment light damage control (LD); C, PBN treated before light damage; D, 4-F-PBN treated before light damage; E, 4-CF_3_-PBN treated before light damage; F, 4-CH_3_-PBN treated before light damage. Two headed arrows showing the thickness of the retinal outer nuclear layer (ONL) that represents the photoreceptor cells. RPE, retinal pigment epithelium; PR, photoreceptors; OPL, outer plexiform layer; INL, inner nuclear layer; IPL, inner plexiform layer; and GCL, ganglion cell layer. The scale bar = 50 μm.
(PDF)

S2 Fig MS-MS analysis using nanospray direct infusion (Advion TriVersa Nanomate) with a Thermo Scientific TSQ Ultra triple quadrupole mass spectrometer. (PDF)

S1 Table Determination of PBN present in the RPE-choroids of baboon. Along with the retinal tissues for rhodopsin assay as described in the methods section, pieces of RPE-Choroid tissue were collected for determination of PBN by mass spectrometry. The amount of PBN was determined by MS-MS analysis as described in S2 Fig. Concentrations were calculated using XCalibur software (Thermo Scientific). PBN determinations in two baboon eyes clearly show that the compound reached the RPE/choroid complex. These results support our finding in mouse eyes treated with PBN, namely that the drug reaches the RPE/choroid complex and inhibits the enzymatic activity of RPE65.
(PDF)
