# Supplementary material for: PBN (Phenyl-N-Tert-Butylnitrone)-Derivatives Are Effective in Slowing the Visual Cycle and Rhodopsin Regeneration and in Protecting the Retina from Light-Induced Damage
Source: PLoS One. 2015 Dec 22;10(12):e0145305. doi: 10.1371/journal.pone.0145305 (PMC4687940; doi:10.1371/journal.pone.0145305)
Supplement: S1 Fig — After ERG recordings, eyes we enucleated for histology, marked for orientation, and fixed. Five-μm sections were cut along the vertical meridian through the optic nerve and stained with H&E. Representative sections from each treatment were imaged from the superior-central retina, which is specifically affected by exposure to damaging light. A, no light damage control (NLD); B, no treatment light damage control (LD); C, PBN treated before light damage; D, 4-F-PBN treated before light damage; E, 4-CF3-PBN treated before light damage; F, 4-CH3-PBN treated before light damage. Two headed arrows showing the thickness of the retinal outer nuclear layer (ONL) that represents the photoreceptor cells. RPE, retinal pigment epithelium; PR, photoreceptors; OPL, outer plexiform layer; INL, inner nuclear layer; IPL, inner plexiform layer; and GCL, ganglion cell layer. The scale bar = 50 μm. (PDF) [file pone.0145305.s001.pdf]

Supplement Information: Figure S1

Stiles et al. : PBN (phenyl-N-tert-butylnitrone)-derivatives are effective in slowing the visual cycle and rhodopsin regeneration and in protecting the retina from light-induced damage.

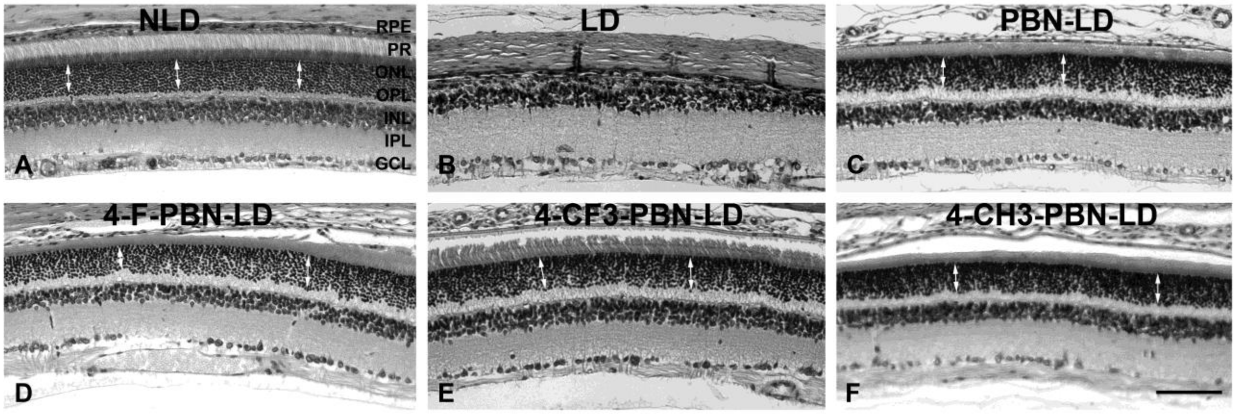

Fig. S1. Representative images of H&E sections of rat eyes treated with PBNDs and light damaged. After ERG recordings, eyes we enucleated for histology, marked for orientation, and fixed. Five-µm sections were cut along the vertical meridian through the optic nerve and stained with H&E. Representative sections from each treatment were imaged from the superior-central retina, which is specifically affected by exposure to damaging light. A, no light damage control (NLD); B, no treatment light damage control (LD); C, PBN treated before light damage; D, 4-F-PBN treated before light damage; E, 4-CF_3-PBN treated before light damage; F, 4-CH_3-PBN treated before light damage. Two headed arrows showing the thickness of the retinal outer nuclear layer (ONL) that represents the photoreceptor cells. RPE, retinal pigment epithelium; PR, photoreceptors; OPL, outer plexiform layer; INL, inner nuclear layer; IPL, inner plexiform layer; and GCL, ganglion cell layer. The scale bar = 50 µm.
