# Supplementary material for: PBN (Phenyl-N-Tert-Butylnitrone)-Derivatives Are Effective in Slowing the Visual Cycle and Rhodopsin Regeneration and in Protecting the Retina from Light-Induced Damage
Source: PLoS One. 2015 Dec 22;10(12):e0145305. doi: 10.1371/journal.pone.0145305 (PMC4687940; doi:10.1371/journal.pone.0145305)
Supplement: S2 Fig — (PDF) [file pone.0145305.s002.pdf]

Supplement Information: Figure S2

Stiles et al. : PBN (phenyl-N-tert-butylnitrone)-derivatives are effective in slowing the visual cycle and rhodopsin regeneration and in protecting the retina from light-induced damage.

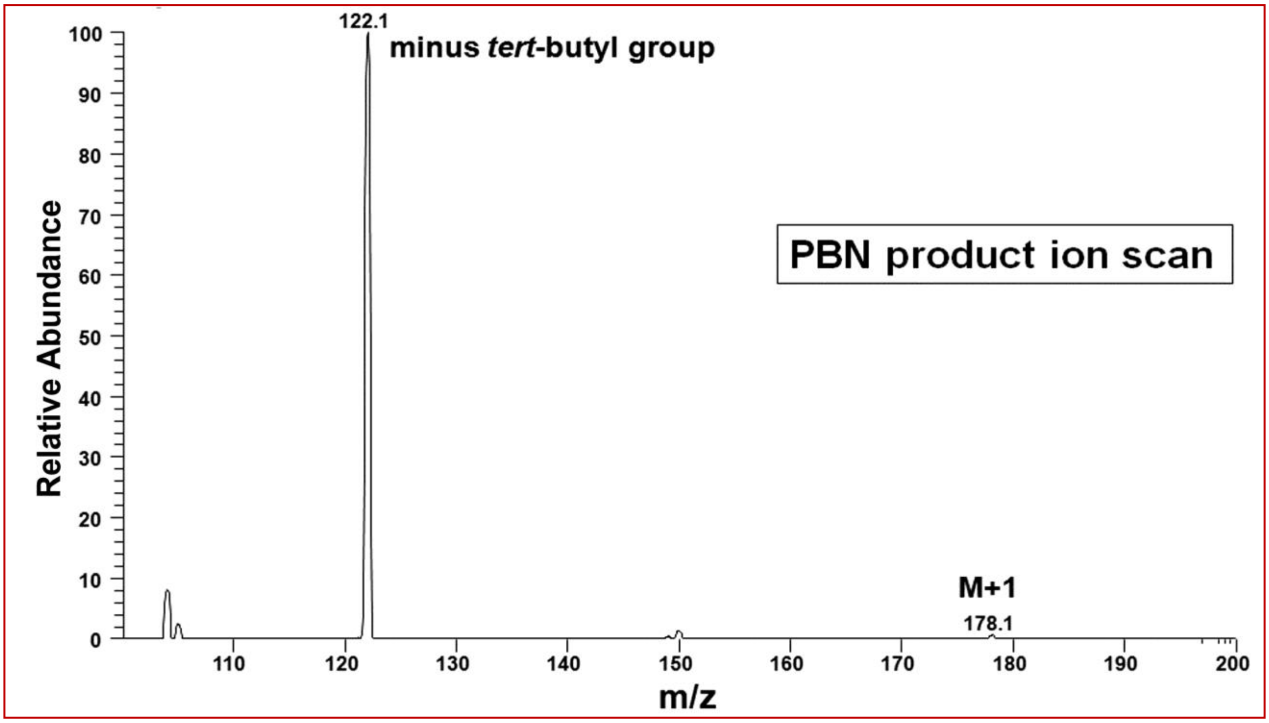

Fig. S2. PBN is detected inside the eye by mass spectrometry- Eyes from mice given one drop of 10% PBN solution or vehicle at 6, 3, and 0 h before euthanasia were removed, washed extensively, and the posterior eye cup extracted and PBN presence determined. Briefly, the RPE/choroid were weighed and homogenized in 40% methanol (aq). The PBN derivative 4-F-PBN was added as an internal standard (0.1 ug). The homogenates were diluted with chloroform:methanol:isopropanol 1:2:4 containing 20 mM ammonium formate. Samples were vortexed, centrifuged, and aliquoted into Eppendorf twin-tec 96-well plates (Fisher Scientific) and sealed with Thermowell sealing tape (Corning). Samples were introduced into a triple quadrupole mass spectrometer (TSQ Ultra, Thermo Scientific) through a chip-based nano-ESI

source (Advion NanoMate) operating in infusion mode. Chipsoft version 8.3.3 software (Advion) was used to set the NanoMate spray voltage to 1.4 kV and the gas pressure to 0.6 psi. The ion transfer tube of the mass spectrometer was maintained at 192 °C. MS/MS spectra were acquired at a rate of 500 m/z per second by methods created using XCalibur software (Thermo Scientific). Analytes were measured in SRM (single reaction monitoring) mode with m/z 178 to 122 for PBN and m/z 196 to 140 for 4-F-PBN. The isolation windows of quadrupoles 1 and 3 were maintained at 0.5 Da, while the collision gas pressure of quadrupole 2 was maintained at 1.0 mtorr of argon. Collision energy was optimized at 20 V. The MS tracing shown in Figure 1 is of PBN found in the eye cup following 3 separate doses. No PBN was found in the eye cups of the vehicle treated control group. The MS result confirms that PBN applied topically reaches the RPE-choroids complex.
