# Supplementary material for: PBN (Phenyl-N-Tert-Butylnitrone)-Derivatives Are Effective in Slowing the Visual Cycle and Rhodopsin Regeneration and in Protecting the Retina from Light-Induced Damage
Source: PLoS One. 2015 Dec 22;10(12):e0145305. doi: 10.1371/journal.pone.0145305 (PMC4687940; doi:10.1371/journal.pone.0145305)
Supplement: S1 Table — Along with the retinal tissues for rhodopsin assay as described in the methods section, pieces of RPE-Choroid tissue were collected for determination of PBN by mass spectrometry. The amount of PBN was determined by MS-MS analysis as described in S2 Fig. Concentrations were calculated using XCalibur software (Thermo Scientific). PBN determinations in two baboon eyes clearly show that the compound reached the RPE/choroid complex. These results support our finding in mouse eyes treated with PBN, namely that the drug reaches the RPE/choroid complex and inhibits the enzymatic activity of RPE65. (PDF) [file pone.0145305.s003.pdf]

Supplement Information: Table 1

Stiles et al. : PBN (phenyl-N-tert-butylnitrone)-derivatives are effective in slowing the visual cycle and rhodopsin regeneration and in protecting the retina from light-induced damage.

Baboon number and treatment	PBN level in RPE/choroid (ng/mg wet weight)
1- Vehicle	0 (Avg of 3 samples from one eye)
1-PBN	24.2 (Avg of 3 samples from one eye)
2-Vehicle	0 (Avg of 4 samples from one eye)
2-PBN	23.5 (Avg of 4 samples from one eye)

Supplement Table 1. Determination of PBN present in the RPE-choroids of baboon. Along with the retinal tissues for rhodopsin assay as described in the methods section, pieces of RPE-Choroid tissue were collected for determination of PBN by mass spectrometry. The amount of PBN was determined by MS-MS analysis as described in the supplement figure 2. Concentrations were calculated using XCalibur software (Thermo Scientific). PBN determinations in two baboon eyes clearly show that the compound reached the RPE/choroid complex. These results support our finding in mouse eyes treated with PBN, namely that the drug reaches the RPE-choroids complex and inhibits the enzymatic activity of RPE65.
